# NF-κB-c-Rel in psoriasis: genetic susceptibility, immunopathogenic circuits, and emerging therapeutic opportunities

**DOI:** 10.3389/fimmu.2026.1849308

**Published:** 2026-07-07

**Authors:** Ruiling Liu, Yujie Wu, Wenjing Jia, Enmei Wang, Shijun J. Zheng, Qingguo Ruan

**Affiliations:** 1 Joint National Laboratory for Antibody Drug Engineering, Henan University School of Medicine, Kaifeng, China; 2 School of Basic Medical Sciences, Henan University School of Medine, Kaifeng, China

**Keywords:** c-Rel, immunomodulation, NF-κB, psoriasis, targeted therapy

## Abstract

Psoriasis is a chronic, relapsing, immune-mediated inflammatory dermatosis characterized by aberrant keratinocyte hyperproliferation and prominent infiltration of inflammatory leukocytes. Among the NF-κB transcription factor family, c-Rel is the first member conclusively implicated as a psoriasis susceptibility gene, and its dysregulated expression positively correlates with clinical disease activity. Mechanistically, c-Rel orchestrates pathogenic inflammation by coordinating immune-cell differentiation programs and inflammatory cytokine production, while also contributing to keratinocyte proliferation and inflammatory responses in non-hematopoietic compartments. Emerging therapeutic approaches that modulate c-Rel, ranging from biologics and small-molecule inhibitors to nucleic-acid-based interventions, have shown encouraging efficacy in both clinical study and experimental psoriasis models. However, key translational hurdles remain, including suboptimal delivery, incomplete target-cell specificity, and long-term safety concerns. Deeper delineation of upstream activation cues and cell-type-restricted c-Rel regulatory circuits will be essential to enable precision targeting of c-Rel in psoriasis.

## 1 Introduction

Psoriasis affects approximately 3% of the global population and represents a prototypical chronic inflammatory skin disease driven by the interplay of genetic predisposition, immune dysregulation, and environmental triggers (1). Psoriasis encompasses multiple clinical subtypes, of which psoriasis vulgaris is the most common, accounting for over 80% of cases (2). Other subtypes include generalized pustular psoriasis, guttate psoriasis, and erythrodermic psoriasis. In addition, psoriasis is associated with a broad spectrum of comorbidities, including psoriatic arthritis, cardiovascular disease, metabolic syndrome, obesity, inflammatory bowel disease, and psychiatric disorders linked to systemic inflammation (3, 4).

Although biologics targeting tumor necrosis factor (TNF)-α, interleukin (IL)-17, and IL-23 have revolutionized psoriasis management, a subset of patients exhibit primary or secondary non-response, and long-term use is associated with increased risk of infections and potential loss of efficacy. Moreover, these agents do not address the upstream transcriptional machinery that coordinates multiple pathogenic pathways. These limitations underscore the need for novel strategies that intercept psoriatic inflammation at a more integrated regulatory level.

The nuclear factor κB (NF-κB) family comprises transcription factors that are fundamental to immunity, inflammation, development, and tumorigenesis, and includes five core members: RelA (p65), RelB, c-Rel, NF-κB1 (p50/p105), and NF-κB2 (p52/p100) (5). Accumulating evidence indicates that the NF-κB family, particularly c-Rel, plays an important role in psoriasis pathogenesis. While broad NF-κB blockade is anti-inflammatory, it is frequently limited by systemic toxicity and impaired host defense. Consequently, attention has shifted toward selective targeting of NF-κB subunits, among which c-Rel has emerged as a particularly compelling node in psoriasis.

## 2 Overview of NF-κB signaling and the distinct biology of c-Rel

NF-κB activation proceeds through canonical and non-canonical pathways, culminating in nuclear translocation of NF-κB dimers and transcriptional induction of inflammatory and survival genes (6, 7). Although NF-κB family members share structural homology, they are not fully redundant. Distinct expression patterns and target-gene repertoires confer cell-type and stimulus specificity. **Table 1** summarizes the functional differences between c−Rel and other NF−κB subunits, with a focus on psoriasis-related inflammatory signaling.

**Table 1:** Distinctions between c-Rel and other NF-κB family members.

Feature	RelA (p65)	RelB	c-Rel	p50 (NF-κB1)	p52 (NF-κB2)
Transactivation domain (TAD)	Yes (C-terminal)	Yes (C-terminal)	Yes (C-terminal)	No (acts as repressor or requires partner)	No (acts as repressor or requires partner)
Primary pathway	Canonical (triggered by TNF, TLR, IL-1)	Non-canonical (triggered by LTβR, BAFF, CD40)	Canonical (also involved in TLR7-specific responses)	Canonical (as p50/p105)	Non-canonical (as p52/p100)
Typical dimerization partner	p50 (p50/p65)	p52 (p52/RelB)	p50 or p65 (c-Rel/p50, c-Rel/p65)	p65, c-Rel, or homodimer (p50/p50)	RelB, or homodimer (p52/p52)
Tissue distribution	Ubiquitous (expressed in most cell types)	Mainly in lymphoid organs, dendritic cells, and macrophages	Enriched in hematopoietic lineages; low in non-immune tissues	Ubiquitous (constitutively processed from p105)	Restricted to lymphoid tissues (processed from p100)
Key biological functions	Master pro-inflammatory transcription factor; regulates anti-apoptotic genes; essential for embryonic development	Regulates dendritic cell maturation, lymphoid organ development; involved in adaptive immunity	T cell activation, Th1/Th17 differentiation, Treg development, B cell proliferation/survival, macrophage cytokine production	DNA-binding subunit; p50/p50 homodimers can repress transcription; p105 has IκB-like function	Similar to p50; p52/RelB drives non-canonical target genes (e.g., CXCL12, ELT)
Roles in psoriasis pathogenesis	Broadly pro-inflammatory, drives TNF-α, IL-6, IL-8, and many acute-phase genes in keratinocytes and immune cells; but its ubiquitous expression limits selective targeting	Not directly implicated; may influence dendritic cell functions in psoriasis indirectly	Central and non-redundant – directly controls IL-23p19, IL-12p40, IL-1β, IL-6, Rorc, Foxp3; links genetic susceptibility (REL variants) to Th17-driven inflammation; also promotes keratinocyte hyperproliferation	p50/p50 homodimers may limit inflammation; p50 haploinsufficiency exacerbates psoriasis-like inflammation; role is context-dependent	Limited evidence; may be involved in lymphoid tissue remodeling in psoriatic arthritis, but not a major driver in cutaneous psoriasis

c-Rel is notable for its enrichment in hematopoietic lineages and comparatively limited expression in most non-immune tissues (8, 9), implying a potentially improved therapeutic window relative to pan-NF-κB inhibition. Accordingly, c-Rel-deficient mice develop normally and retain effective or only mildly diminished resistance to viral and bacterial infections (10–14). Functionally, c-Rel is a signal-dependent regulator of immune activation, inflammatory cytokine production, and T cell fate decisions, particularly within T helper (Th)1, Th17, and regulatory T cell (Treg) compartments. Consistent with this immunological specialization, c-Rel has been implicated in multiple inflammatory and autoimmune disorders, including psoriasis (15).

## 3 c-Rel as a psoriasis susceptibility gene

Genetic studies—including family aggregation, twin concordance, and genome-wide association studies (GWAS)—underscore psoriasis heritability. Notably, concordance rates range from 35% to 70% in monozygotic twins, compared with 12% to 23% in dizygotic twins (16). GWAS have identified more than 50 psoriasis susceptibility genes, spanning innate immunity (e.g., CARD14, c-REL, TRAF3IP2, IFIH1, and DDX58), antigen presentation (e.g., HLA-Cw6, ERAP1, and ERAP2), T-cell development, maturation, and differentiation (e.g., RUNX1, RUNX3, and STAT3), adaptive immunity (e.g., HLA-Cw6, ERAP1/2, and RUNX1/3), cytokine signaling (e.g., IL-12p40, IL-23p19, IL-23R, and JAK2), and immune regulatory factors (e.g., TNIP1, TNFAIP3, IL36RN, SOCS1, ZC3H12C, and NFKBIA) (1). The strongest genetic effect localizes to chromosome 6p21.3 within the major histocompatibility complex (MHC) region and accounts for approximately one-third of the heritable contribution to disease risk. Within this region, HLA-Cw6 (the PSORS1 locus) represents the most prominent susceptibility factor (present in >50% of patients) (17, 18). Environmental factors also contribute to triggering or exacerbating psoriasis, particularly in genetically predisposed individuals. For example, obesity is commonly considered a major risk factor for psoriasis (3, 16). In addition, individuals with one autoimmune disease are more likely to develop others, and patients with psoriasis have increased prevalences of rheumatoid arthritis, celiac disease, and Crohn’s disease (19).

Only a subset of NF-κB family members has been implicated in psoriasis susceptibility, and c-Rel was the first NF-κB family member to be clearly established as a psoriasis risk gene. The REL gene maps to the 6p21-p22 region, which is enriched for psoriasis susceptibility loci. Population studies have repeatedly linked the REL variant rs702873 to increased psoriasis risk, particularly in European and Asian cohorts, with risk alleles associated with enhanced c-Rel expression or activity (20, 21). Importantly, c-Rel is elevated in psoriatic lesional skin and decreases with effective therapy, supporting a model wherein genetic predisposition and inflammatory microenvironment synergize to enforce c-Rel hyperactivation (5). In contrast, stable associations for RelA, RelB, and NF-κB2 have not been consistently demonstrated, although transcriptome-wide association study (TWAS) suggests additional NF-κB-related genes (e.g., NF-κB1; NFKBIA) may contribute to disease susceptibility (21, 22). **Table 2** lists psoriasis susceptibility genes linked to the NF−κB/c−Rel signaling cascade, encompassing c−Rel, its downstream target genes, and core regulatory factors governing NF−κB signaling. Comprehensive details regarding variant classifications, studied cohorts, functional consequences, and mechanistic associations with the NF−κB/c−Rel signaling axis are documented in the table.

**Table 2:** Psoriasis susceptibility genes associated with NF-κB/c-Rel signaling.

Gene	Variant(s)	Population/study design	Functional consequence & relationship to NF-κB/c-Rel
REL (c-Rel)	rs702873, rs13017599	European, Asian; meta-analysis	Encodes c-Rel subunit itself, associated with increased c-Rel expression/activity
TNIP1	rs17728338 (A>G), rs3762999, rs999556	European, Chinese Han	Encodes ABIN1, a negative regulator of NF-κB
		(13,908 cases, 20,051 controls)	
NFKBIA	rs12884468, rs12883343	European; Chinese	Encodes IκBα, which sequesters NF-κB in cytoplasm
		(psoriatic arthritis vs. cutaneous psoriasis)	
TNFAIP3	rs610604 (G>T), rs582757, rs6918329	Multiple populations; European (4,704 cases, 7,805 controls)	Encodes A20, a deubiquitinase that terminates NF-κB signaling
CARD14	Gain-of-function: p.Gly117Ser, p.Glu138Ala;	European, Taiwanese, Chinese Han	Encodes a scaffold protein, which activates NF-κB signaling
	rare missense variants		
IL12B	rs3212227 (3′UTR), rs6887695	Multiple populations; large-scale GWAS	Encodes p40, shared subunit of IL-12 and IL-23. c-Rel directly binds κB elements in the IL12B promoter and drive its expression
IL23A	rs2066808	European; UK/Ireland PsA cohort	Encodes p19, the IL-23-specific subunit. c-Rel directly binds the c-Rel recognition site within the IL23A promoter and drive its expression
IL23R	Multiple SNPs (e.g., rs11209026, rs7530511)	European; GWAS	Encodes the IL-23 receptor. Functions downstream of c-Rel-driven IL-23 production.
NFKB1	rs1020760, rs1609798	European; sequencing-based approach	Encodes p105/p50, indirectly altering NF-κB dimer composition and c-Rel dependent transcriptional output.
RNF114	rs495337	European; multiple GWAS	Encodes a ubiquitin ligase regulating T-cell activation, modulates NF-κB signaling through ubiquitination pathways, potentially influencing c-Rel activity indirectly.

## 4 Mechanistic roles of c-Rel in psoriasis pathogenesis

**Figure 1:**
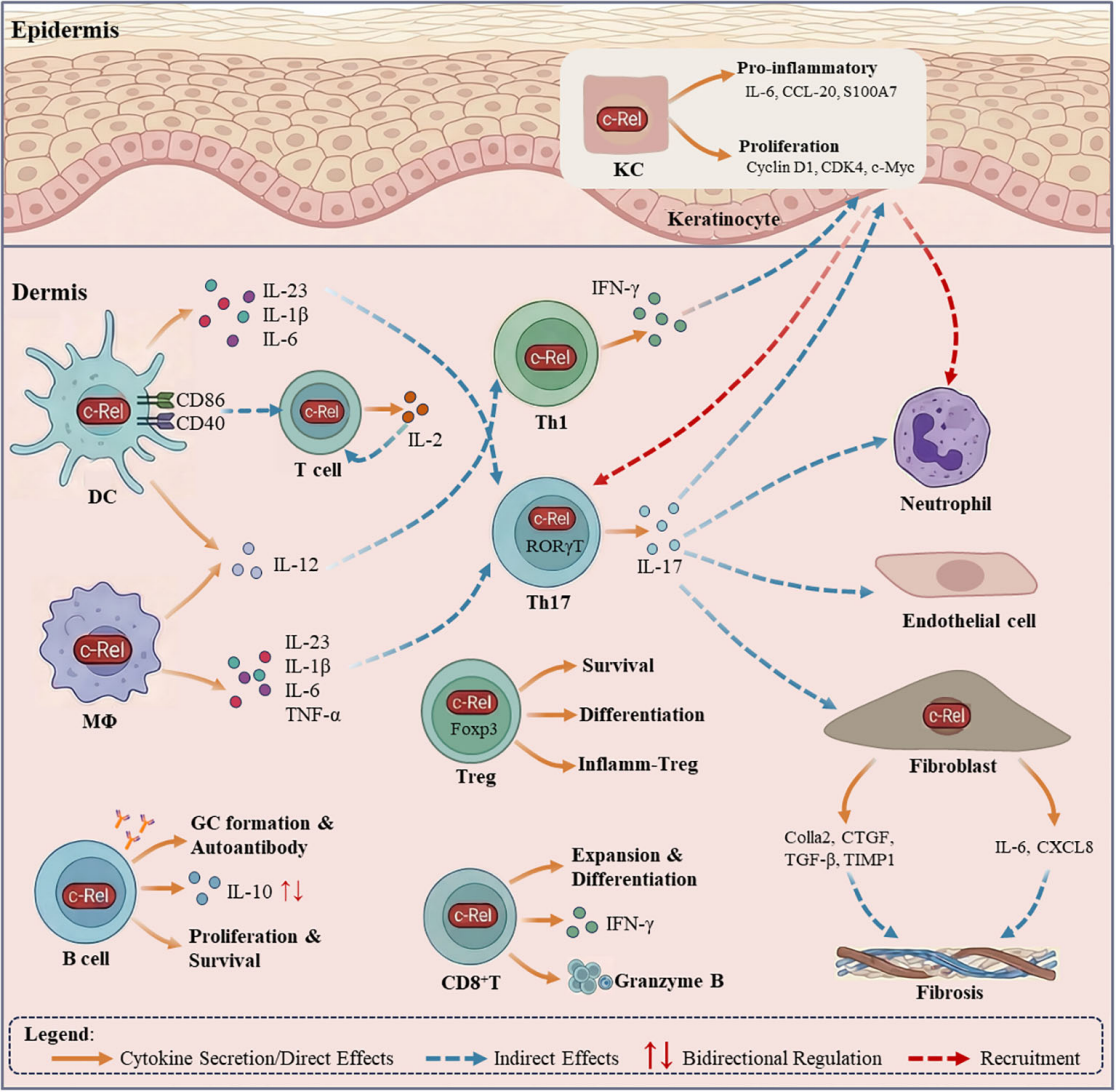
The comprehensive effects of c-Rel on psoriasis pathogenesis. c-Rel orchestrates pathogenic inflammation in psoriasis by coordinating immune-cell differentiation programs and inflammatory cytokine production, while also contributing to keratinocyte proliferation and inflammatory responses in non-hematopoietic compartments. DC, Dendritic Cell; MΦ, Macrophage; KC, Keratinocyte; Inflamm-Treg, Inflammatory Regulatory T Cell.

Psoriasis pathogenesis is commonly conceptualized as a self-sustaining inflammatory loop centered on a dendritic cell (DC)-T cell-keratinocyte “pathogenic triangle” (23, 24). Triggering events (mechanical stress, infection, drugs) induce danger signals that activate keratinocytes and innate immune cells, initiating cytokine cascades that promote DC maturation and T cell polarization (25, 26). Effector cytokines (notably IL-17, IL-22, and IFN-γ) then act on keratinocytes to drive hyperproliferation and chemokine secretion, recruiting additional leukocytes and perpetuating chronic inflammation. Innate immune cells are key pathogenic drivers in early psoriasis, whereas T cell-dominated adaptive immunity is a hallmark of later stages. Within this framework, c-Rel functions as a transcriptional amplifier at multiple immunological checkpoints. In addition, emerging evidence indicates that c-Rel expression in non-immune cells also play important roles in psoriasis pathogenesis. The comprehensive effects of c−Rel on psoriasis pathogenesis are summarized in **Figure 1**. Of note, we focus solely on cell populations whose functions are both directly modulated by c−Rel and implicated in the development of psoriasis.

### 4.1 c-Rel in immune cells: amplifying the psoriatic inflammatory circuit

#### 4.1.1 Dendritic cells

Distinct DC subsets play markedly different yet highly synergistic roles in psoriasis. While plasmacytoid DCs (pDCs) are responsible for disease initiation (27), inflammatory DCs sustainably drive Th17 cells via IL−23 (28). In contrast, Langerhans cells (LCs) exert negative regulatory effects (27). c-Rel regulates CD86 and CD40 expression on DCs and thereby modulating their antigen-presenting function (29). In addition, c-Rel is critical for Toll-like receptor (TLR)-driven DC maturation and pro-inflammatory cytokine transcription. In TLR7-activated DCs, c-Rel predominantly forms heterodimers with p65 to drive transcription of IL-23, IL-12, IL-1β, and IL-6 (30), thereby promoting Th17 and Th1 differentiation, both of which are critical in psoriasis pathogenesis. In addition, IL-23 is a key signal that activates γδ T cells in the skin and promotes their production of IL-17 (31). IL-1β and IL-23 produced by DCs can activate Type 3 innate lymphoid cells (ILC3) and stimulate its secretion of IL-22, which is involved in epidermal hyperplasia and the initiation of inflammation in the early stage of psoriasis (32). Selective inhibition of c-Rel disrupts these c-Rel/p65 heterodimers, shifting the balance toward p50/p65 or p65/p65 dimers, which have different promoter affinities and transcriptional outputs. Consequently, TLR7-mediated production of IL-23, IL-12, and IL-1β is markedly reduced, while certain homeostatic NF-κB target genes remain intact. This dimer-switching mechanism explains the selective anti-inflammatory effect of c-Rel inhibition in TLR7-driven psoriatic inflammation.

#### 4.1.2 Macrophages

Activated macrophages increase in psoriatic lesions and are a major source of pro-inflammatory cytokines (33). They can also directly shape the pathogenicity of T cells and play an indispensable role in sustaining and amplifying skin inflammation. Depletion of macrophages alleviates psoriatic skin inflammation (33). c-Rel is selectively required for inducible transcription of IL-12/IL-23 (p40) (34), a shared subunit central to both Th1 and Th17 pathways. By controlling p40 output, c-Rel can gate the magnitude of IL-23-driven Th17 responses and concomitant production of IL-6, TNF-α, and IL-1β. Macrophages represent a major source of IL-23 in lesional skin, and inhibiting c-Rel can selectively block macrophage IL-23 synthesis, thereby dampening Th17-driven immune responses. c-Rel in macrophages can also activate γδT cells and group 3 innate lymphoid cells (ILC3) by promoting the secretion of IL-23 and IL-1β, a process that also occurs in DCs.

#### 4.1.3 T cells

T cells play a central role in psoriasis pathogenesis. In a mouse model of spontaneous psoriasiform dermatitis, researchers found that depletion of CD4^+^ but not CD8^+^ T cells led to complete resolution of dermatitis (35). However, it has also been reported that CD8^+^ T cells were essential for psoriatic skin lesions using a humanized psoriasis mouse model (36). c−Rel is a core regulator of T cell activation, subset differentiation, proliferation, survival, and functional modulation. It directly regulates the chromatin remodeling of the promoters of key genes such as IL-2 and GM-CSF to initiate transcription. In addition, it critically modulates the differentiation of CD4^+^ T subsets (including Th1, Th17, and Treg cells) and governs CD8^+^ T cell effector function during the pathogenesis of psoriasis.

##### 4.1.3.1 Th17 cells

Studies have found that the number of Th17 cells in the peripheral blood and skin lesions of psoriasis patients is significantly higher than that in healthy individuals (37). As an early inflammatory activator, IL-17 stimulates keratinocytes, endothelial cells, mast cells, and fibroblasts to produce inflammatory cytokines (38, 39). In addition, IL-17 is one of the core cytokines that recruit neutrophils (40). c-Rel is a core transcriptional regulator of Th17 differentiation. Our studies showed that, in response to IL-23, IL-6, and TGF-β, c-Rel binds the Rorc promoter to upregulate RORγt, thereby enabling robust expression of IL-17 and IL-22 (41).

##### 4.1.3.2 Th1 cells

Similar to Th17 cells, the proportion of Th1 cells is also significantly increased in the peripheral blood of patients with psoriasis (37). Th1 cells amplify cutaneous inflammation primarily by secreting IFN−γ, which activates keratinocytes and induces the production of chemokines (e.g., CXCL9/10). This leads to the recruitment of additional immune cells to inflammatory sites and further aggravating keratinocyte hyperplasia (42). Moreover, IFN-γ can stimulate myeloid antigen-presenting cells (APCs) to upregulate IL-1β and IL-23, thereby promoting Th17 differentiation and facilitating their recruitment to psoriatic lesions (39, 43). c-Rel is a central regulator of Th1 differentiation and is indispensable for Th1-driven autoimmune inflammation. c-Rel directly promotes IFN-γ transcription and is essential for Th1 effector function. IFN-γ expression is abrogated in c-Rel-deficient T cells (44, 45).

##### 4.1.3.3 Treg cells

Treg cells play a crucial role in maintaining immune homeostasis in psoriasis by suppressing the abnormal activation of pathogenic T cells and reducing the production of pro-inflammatory cytokines, thereby alleviating skin inflammation and preventing disease progression. Findings regarding whether Treg levels are increased or decreased in psoriasis remain conflicting, which may depend on disease stages and sampling sites. Notably, most studies have confirmed that Treg function is impaired in patients with psoriasis (46). c-Rel plays a dual role in the development and function of Tregs. It is required for thymic Treg (tTreg) lineage commitment by activating Foxp3 through conserved non−coding sequence 3 (CNS3) enhancer engagement and promotes the survival of thymic Treg precursors through the up-regulation of anti-apoptotic genes (BCL-2 and BCL-xL) and IL-2R (47–49). Conversely, our work showed that, within inflammatory microenvironments, c-Rel upregulation in Tregs may promote inflammatory reprogramming and diminished suppressive function. c-Rel inhibition in this context can restore immunoregulatory capacity (50).

##### 4.1.3.4 CD8 cells

CD8^+^ cytotoxic T cells directly attack the epidermis and amplify local inflammatory responses. The abundance of CD8^+^ T cell subsets is markedly increased in lesional skin (51). CD8^+^ tissue-resident memory T cells are predominantly localized in the epidermis and represent a key driver of recurrent flares in clinically resolved psoriatic skin (52). c−Rel acts as a critical transcription factor in regulating the activation, proliferation, and effector functions of CD8^+^ T cells. Upon antigen stimulation, c−Rel promotes the expression of key effector molecules including IL−2, IFN−γ, and granzyme B, thereby enhancing the cytotoxic activity of CD8^+^ T cells (53–55). Additionally, c−Rel supports the clonal expansion and differentiation of CD8^+^ T cells into effector and memory subsets by maintaining cell survival and regulating metabolic reprogramming (55, 56).

#### 4.1.4 B cells

B cells play a dual role in psoriasis rather than simply promoting or inhibiting psoriasis pathogenesis. On one hand, increased total B cell numbers have been observed in some patients and in local skin lesions, suggesting their involvement in psoriatic inflammation. B cells may contribute to inflammatory processes by producing autoantibodies and proinflammatory cytokines, forming ectopic lymphoid follicles in the skin (57) or trigger the T-cell-mediated autoimmune response against melanocytes (58). On the other hand, regulatory B cells (Bregs) can alleviate psoriasis by secreting IL-10 (59, 60). c-Rel in B cells plays a critical role in psoriasis pathogenesis by regulating B cell function and immune crosstalk. As a key transcription factor, c-Rel promotes germinal center formation, autoantibody production (61). In addition, c-Rel plays essential roles in activated B cell growth, proliferation, and survival through the induction of antiapoptotic MYC and BCL2L1 (15, 62). c-Rel also regulates Breg function. c-Rel-deficient B cells produce less IL-10 upon BCR stimulation (63). In contrast, c-Rel restricts IL-10 production in Bregs via inhibiting Blimp-1 under TLR stimulation (e.g., CpG), preventing excessive immunosuppression (64). These findings indicate that the regulation of IL-10 production in B cells by c-Rel is probably stimulus-dependent.

### 4.2 c-Rel in non-hematopoietic compartments: linking immunity to epidermal hyperplasia and tissue remodeling

#### 4.2.1 Keratinocytes

Keratinocytes serve as indispensable participants and amplifiers in the pathogenesis of psoriasis (65). They exhibit hyperproliferation and increased Ki-67 expression in psoriasis. Environmental factors (e.g., stress, infection, trauma) activate keratinocytes to release antimicrobial peptides such as LL-37, which form complexes with self-DNA/RNA and activate pDCs (66). Keratinocytes produce CXCL1/2/8 and CCL20 in response to IL-17, recruiting neutrophils and CCR6^+^ cells (38) Lesional keratinocytes exhibit increased c-Rel expression. The proliferative capacity of keratinocyte from c-Rel-deficient mice is significantly reduced and the epidermis becomes thinner, supporting a role for c-Rel in controlling keratinocyte proliferation (5). In addition, c-Rel directly transactivates cell-cycle drivers (Cyclin D1, CDK4, and c-Myc), accelerating G1/S progression and contributing to epidermal hyperplasia and plaque formation (67, 68). However, in contrast to animals lacking c-Rel alone, mice lacking both p65 and c-Rel developed severe dermatitis (69).

#### 4.2.2 Fibroblasts

Psoriasis is primarily regarded as an inflammatory epidermal disease, but sustained Th1− and Th17−driven inflammation activates dermal fibroblasts, accelerates collagen deposition, and promotes mild cutaneous fibrosis. In turn, fibrotic remodeling stabilizes immune infiltration, amplifies chronic inflammation, and contributes to lesion thickening and recurrence in progressive psoriasis (70, 71). c-Rel activation in fibroblasts induces pro-fibrotic programs (e.g., COL1A2, CTGF, TGF-β, TIMP1) and inflammatory mediators (IL-6, CXCL8), promoting extracellular matrix deposition and dermal remodeling (72, 73). Genetic loss of c-Rel attenuates bleomycin-induced skin fibrosis (5), suggesting that c-Rel may contribute to psoriatic skin remodeling by regulating fibroblast activation.

Notably, while γδT, ILC3, mast cells, endothelial cells, melanocytes, and Schwann cells all play important roles in the pathogenesis of psoriasis, direct evidence is currently lacking that c-Rel directly regulates the function of these cells and thereby influences the development of psoriasis.

## 5 Therapeutic targeting of c-Rel in psoriasis: current strategies and evidence

### 5.1 Rationale: why c-Rel represents a high-value target

Because the NF-κB family regulates expression of numerous genes involved in inflammation and immune responses, NF-κB has long been considered a classic molecular target for autoimmune disease therapy. However, conventional approaches for NF-κB suppression (e.g., glucocorticoids, thalidomide) can be effective but are limited by lack of specificity and systemic adverse effects (74, 75). This is because NF-κB family proteins are expressed in many cell types and are required for normal physiological processes, including regulation of innate and adaptive immunity during infection, anti-apoptotic programs, and cell proliferation (8). As a result, although agents such as Dehydroxymethylepoxyquinomicin (DHMEQ, an inhibitor that primarily targets p65), SN50 (blocks nuclear translocation of p50/p65 heterodimers), and BAY 11-7082 (a pan-NF-κB inhibitor) have shown anti-inflammatory effects in certain autoimmune models, their broader inhibition of canonical NF-κB signaling may increase the risk of systemic toxicity and infection, further supporting the rationale for subunit-selective strategies centered on c-Rel (74). Upstream/downstream blockade (e.g., TNF inhibitors; IL-17/IL-23 inhibitors) has transformed clinical care yet leaves a subset of patients with incomplete responses, loss of efficacy, or intolerance (76, 77). Selectively targeting c-Rel offers a conceptually attractive middle path: dampening pathogenic inflammatory circuits while sparing broader NF-κB functions essential for tissue homeostasis and antimicrobial defense (78).

### 5.2 Representative modalities

**Figure 2:**
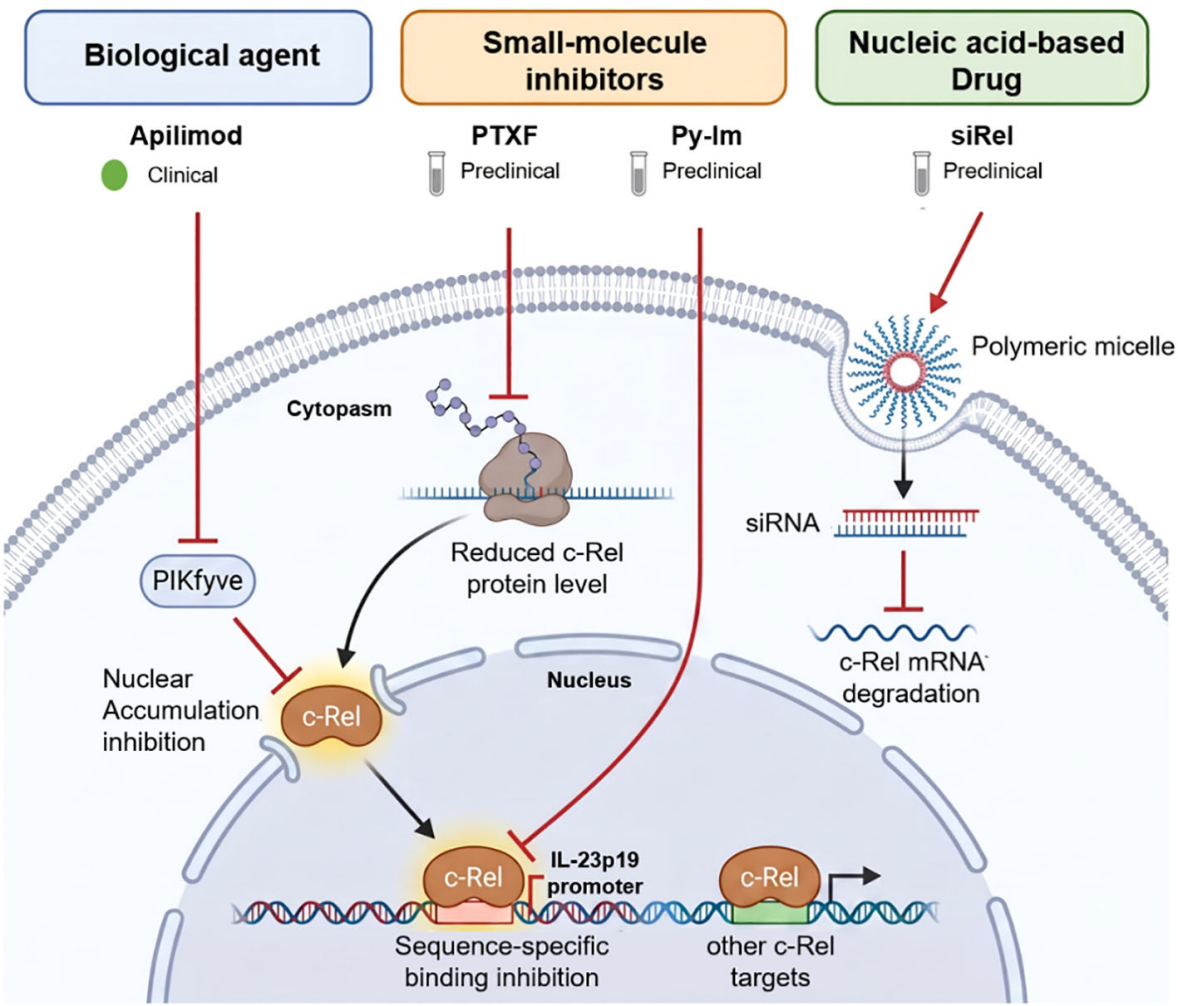
Schematic overview of c-Rel-targeted therapeutic strategies for psoriasis. This schematic summarizes three major classes of agents that interfere with c−Rel function in psoriasis, including biologic agent (Apilimod), small molecule inhibitors (Pentoxifylline and pyrrole−imidazole polyamide), and nucleic acid−based drug (c−Rel−targeting siRNA). The net consequence of these interventions is decreased c−Rel−dependent expression of IL−23, IL−12, IL−1β and IL−6, leading to impaired Th17/Th1 differentiation, reduced keratinocyte hyperproliferation and attenuation of psoriatic inflammation. PIKfyve, phosphatidylinositol−3−phosphate 5−kinase; PTXF, pentoxifylline; Py−Im, pyrrole−imidazole; siRNA, small interfering RNA; siRel, c−Rel−targeting siRNA.

Current c−Rel-targeted therapeutic strategies for psoriasis fall into three major categories, which are outlined in **Figure 2**.

#### 5.2.1 Biological agent

Apilimod, an orally available agent initially described as an IL-12/IL-23 inhibitor. Mechanistically, it reduces c-Rel nuclear accumulation (with minimal impact on p65/p50) and downregulates the transcription of IL-12p35, IL-12/IL-23p40, and IL-23p19 (79). In a clinical study involving patients with moderate-to-severe psoriasis, oral apilimod reduced the expression of IL-12/IL-23 and decreased DC infiltration in lesional skin. This study provided proof-of-concept that pharmacological reduction of c-Rel nuclear accumulation is achievable in humans and correlates with clinical improvement. However, the study was limited by a small sample size and the absence of long-term safety data. No serious adverse events were reported, but mild gastrointestinal symptoms were noted (79). Nevertheless, marked histological improvements were observed among psoriasis patients enrolled in this open-label Phase 2a clinical trial.

#### 5.2.2 Small-molecule inhibitors

##### 5.2.2.1 Pentoxifylline

PTXF is a non-specific phosphodiesterase inhibitor with FDA-approved indications (80). It exerts anti-inflammatory effects and suppresses c-Rel-associated cytokines, including TNF-α, IL-6, and IL-17, without substantially affecting RelA expression (81, 82). Topical PTXF-containing compounded formulations have shown favorable stability and clinical activity in mild-to-moderate plaque psoriasis, with acceptable safety profiles (83). Other studies have reported that PTXF combined with Yinxieling ointment yields satisfactory outcomes in psoriasis vulgaris, including higher response rates than in control groups (84). However, PTXF may also impair the suppressive function of Treg cells, raising concerns regarding long-term immunological consequences of sustained c-Rel pathway interference (48).

Currently, PTXF is predominantly administered as an add-on therapeutic agent.

##### 5.2.2.2 Gene-silencing pyrrole-imidazole polyamides

We have developed a Py-Im polyamide that binds the c-Rel recognition sequence within the IL-23p19 promoter. This agent selectively suppresses IL-23 expression without broadly inhibiting other c-Rel target genes (85). In Imiquimod (IMQ) models, such agent reduced erythema, scaling, and thickening in a dose-dependent manner and attenuate inflammatory infiltrates. These findings suggest that Py-Im polyamides may provide a highly specific and potentially non-immunogenic alternative to antibody-based approaches. However, several obstacles remain before the clinical translation of such agent, including insufficient tissue penetration and uncharacterized off−target risks.

#### 5.2.3 Nucleic-acid-based therapeutics

siRNA therapeutics offer a promising strategy for disease treatment by selectively silencing pathogenic genes through the RNA interference pathway. This approach reduces disease-related protein expression and may expand therapeutic options for otherwise difficult-to-treat disorders. We developed a c-Rel-targeting siRNA for psoriasis intervention. After chemical stabilization and delivery using polymeric micelle systems such as PEG-PLL-PLLeu, this siRNA reduced c-Rel expression *in vivo*. It also prevented and treated IMQ-induced psoriasiform inflammation (86). These interventions decreased IL-23p19, IL-17A, IL-1β, and IL-6 and mitigate epidermal hyperplasia. However, delivery hurdles constitute the primary bottleneck, including enzymatic degradation, short half-life and poor delivery efficiency toward target immune cell subsets. Alternative carrier systems, including exosomes and Distearoylphosphatidylserine (DSPS)-bearing lipid nanoparticles, are being explored to boost cellular uptake in immune cells while lowering toxic risks (87, 88). Additional limitations include off-target RNAi effects and skin-barrier-mediated hindrance of topical drug delivery.

#### 5.2.4 Other c-Rel-specific inhibitors that have not been applied to psoriasis treatment

Additional inhibitors that can substantially suppress c-Rel activity have been reported, but they have not yet been applied to psoriasis treatment.

R96A is a c-Rel-selective small-molecule inhibitor that binds the Rel homology domain. Through this interaction, it inhibits both c-Rel nuclear translocation and DNA binding. Although primarily investigated in myeloid immunoregulation (89) and other autoimmunity models (e.g., Experimental Autoimmune Encephalomyelitis), its mechanistic profile suggests that it could serve as a candidate scaffold for future psoriasis-directed drug development.

Three synthetic epoxyquinone compounds, namely jesterone dimer (JD), epoxyquinone A monomer (EqM), and calafianin monomer (CM101), induce apoptosis in IκBα-deficient lymphoma cells by directly suppressing c-Rel DNA binding activity across multiple human lymphoma cell lines (90, 91). Although these compounds have not been used in psoriasis treatment, their mechanistic relevance suggests potential future applications.

CRISPR/Cas9-mediated, DC-specific knockout of c-Rel selectively suppresses TLR7-induced inflammation. Notably, it does not appear to affect inflammatory pathways mediated by TLR3 or TLR9. It also markedly reduces the ability of c-Rel to promote Th17 differentiation (30). This targeting advantage makes it a potential direction for cell-based therapy in psoriasis. However, the specificity and safety of this gene-editing strategy require further validation, and the work remains at a preclinical stage.

## 6 Key challenges for clinical translation

### 6.1 c-Rel suppression approaches

Because c-Rel is a nuclear transcription factor, effective inhibition requires intracellular delivery and often nuclear access. Small molecules may achieve this but can carry off-target toxicity. Conversely, siRNA approaches are constrained by enzymatic degradation, short half-life, and inefficient delivery to relevant immune subsets, necessitating optimized chemistry and carrier systems (92).

### 6.2 Delivery and tissue targeting

Improving the efficiency and stability of c-Rel inhibitor delivery into immune cells is an urgent challenge for clinically targeting c-Rel to treat inflammation-related diseases. For cutaneous disease, topical administration is attractive but must overcome the skin barrier and ensure uptake by pathogenic immune cells within lesional skin. Advanced carriers such as exosomes and engineered lipid nanoparticles may improve stability, cell targeting, and penetration. For example, mesenchymal stem cell-derived exosomes loaded with c-Rel siRNA show superior efficacy to nanopolymers in promoting corneal wound healing (87). In addition, lipid nanoparticles (LNPs) containing DSPS can be used for skin-related delivery. These particles show low overall cytotoxicity and can sustain c-Rel suppression over an extended period (88). On the other hand, viral vectors have been evaluated as effective delivery vehicles for various diseases. Our study showed that AAV6-mediated delivery of c-Rel-specific shRNA (AAV6-shRel) achieved c-Rel suppression *in vivo*, significantly prolonged corneal graft survival (82). However, while viral vectors provide efficient transduction and durable silencing, they give rise to additional considerations related to immunogenicity, the control of biodistribution, and regulatory complexity.

### 6.3 Long-term safety and disease modeling limitations

Short-term c-Rel inhibition appears well tolerated in multiple preclinical settings, consistent with the relatively restricted basal expression of c-Rel in non-immune tissues. However, it has been reported that inherited c-Rel deficiency in humans disrupts the development and function of multiple myeloid and lymphoid cells, compromising innate and adaptive immunity to multiple infectious agents (29), through current c-Rel-suppressing approaches may not result in complete c-Rel deficiency. In addition, chronic suppression could disrupt immune homeostasis, alter Treg biology, and increase infection or malignancy risk. Moreover, the IMQ model largely reflects acute psoriasiform inflammation rather than the full complexity of chronic human plaque psoriasis (93, 94), underscoring the need for diversified models and long-term evaluation.

## 7 Discussion

Targeting c-Rel represents a mechanistically grounded strategy to intercept psoriasis at the level of transcriptional control over autoimmune response and keratinocyte hyperproliferation. Future progress will likely depend on: (1) mapping upstream activators of c-Rel in cell-type-specific contexts, particularly the details of its interplay with TLR7 signaling and the coordinated regulatory networks between immune and non-immune cells; (2) engineering delivery systems that achieve lesion-restricted, immune-cell-selective inhibition with minimal systemic exposure, and (3) conducting robust, long-term clinical studies to define efficacy, durability, and safety, including effects on comorbidities such as psoriatic arthritis. Finally, integration of GWAS-informed risk stratification with therapeutic decision-making may enable precision immunomodulation, positioning c-Rel inhibition either as a stand-alone option for selected patients or as a rational component of multi-target combination regimens.
